# Effectiveness of the Alfalfa App in Warfarin Therapy Management for Patients Undergoing Venous Thrombosis Prevention and Treatment: Cohort Study

**DOI:** 10.2196/23332

**Published:** 2021-03-02

**Authors:** Hua Cao, Shaojun Jiang, Meina Lv, Tingting Wu, Wenjun Chen, Jinhua Zhang

**Affiliations:** 1 Department of Cardiac Surgery Fujian Medical University Union Hospital Fuzhou China; 2 Department of Cardiac Surgery Fujian Maternity and Children Health Hospital Fuzhou China; 3 Department of Pharmacy Fujian Medical University Union Hospital Fuzhou China; 4 College of Pharmacy Fujian Medical University Fuzhou China

**Keywords:** warfarin, anticoagulation, smartphone, telemedicine, app, online, offline, bleeding, INR, TTR, mobile phone

## Abstract

**Background:**

Over the years, the internet has enabled considerable progress in the management of chronic diseases, especially hypertension and diabetes. It also provides novel opportunities in online anticoagulation management. Nevertheless, there is insufficient evidence regarding the effectiveness of online anticoagulation management.

**Objective:**

This study explored the effectiveness and safety of warfarin management via the Alfalfa app, so as to provide evidence in support of anticoagulant management through online services.

**Methods:**

In this retrospective, observational cohort study, 824 patients were included. In the offline group, patients went to the hospital clinic for warfarin management. In the Alfalfa app group, patients reported the dose of warfarin, current international normalized ratio (INR) value, and other related information through the Alfalfa app. Physicians or pharmacists used the app to adjust the dose of warfarin and determined the time for the next blood INR testing. Patients completed INR testing by point-of-care at home or hospital. The primary outcome of the study was the percentage of time in therapeutic range (TTR). Secondary outcomes included minor and major bleeding events, thrombotic events, warfarin-related emergency department visits, hospital admissions, and high INR values.

**Results:**

The TTR and percentage of INR values in the range were significantly higher in the Alfalfa app group than in the offline group (79.35% vs 52.38%, *P*<.001; 3314/4282, 77.39% vs 2005/4202, 47.72%, *P*<.001, respectively). Patients managed via the Alfalfa app had lower rates of subtherapeutic (172/4282, 4.02% vs 388/4202, 9.23%; *P*<.001), supratherapeutic (487/4282, 11.37% vs 882/4202, 20.99%; *P*<.001), and extreme subtherapeutic INR values (290/4282, 6.77% vs 910/4202, 21.66%; *P*<.001). Additionally, the Alfalfa app group had lower incidences of major bleeding (2/425, 0.5% vs 12/399, 3.0%; *P*=.005), warfarin-related emergency department visits (13/425, 3.1% vs 37/399, 9.3%; *P*<.001), and hospital admissions (1/425, 0.2% vs 12/399, 3.0%; *P*=.001) compared with the offline group. However, the Alfalfa app group had a higher incidence of minor bleeding than the offline group (45/425, 10.6% vs 20/399, 5.0%; *P*=.003). There were similar incidences in extreme supratherapeutic INR values (19/4282, 0.44% vs 17/4202, 0.40%; *P*=.78) and thromboembolic events (1/425, 0.2% vs 1/399, 0.3%; *P*=.53) between the two groups.

**Conclusions:**

Warfarin management is superior via the Alfalfa app than via offline services in terms of major bleeding events, warfarin-related emergency department visits, and hospital admissions.

## Introduction

Venous thrombosis, which includes deep vein thrombosis, pulmonary embolism, and cardiogenic stroke, poses a significant burden on the health care system [1]. Anticoagulants are effective in the treatment of venous thrombosis. Patients with atrial fibrillation and valve replacement surgery are at high risk for venous thrombosis; therefore, these patients require anticoagulant treatment. Warfarin is the most commonly used oral anticoagulant due to its low price, specific antagonists, and substantial evidence-based clinical data [2]. The dose of warfarin required for anticoagulation is closely related to dietary vitamin K intake, concurrent medications, the presence of other diseases, body weight, and aerobic exercise. Therefore, warfarin doses during anticoagulation therapy need to be adjusted according to the results of international normalized ratio (INR) tests. Without proper adjustment, excessive warfarin may cause bleeding, and inadequate warfarin doses may cause thrombosis and possible death [3]. This represents a costly use of medical resources and is physically and psychologically traumatic to patients and their families [4].

Warfarin dose adjustment is usually performed at hospital clinics. For patients living in rural areas, adjustment of warfarin dosages requires transportation, accommodation, and time. Patients with low incomes are willing to risk thrombosis and bleeding in an attempt to reduce costs. Online services such as smartphones, text messages, Bluetooth, and communication platforms may assist in adjusting warfarin doses [4-6]. Our meta-analysis, which included 16,915 patients, of whom 8655 had their warfarin dosage adjusted in the hospital and 8260 had their warfarin dosages adjusted through online services, revealed that online services were associated with fewer warfarin-related hospital admissions than hospital management (odds ratio 0.47, 95% CI 0.30-0.73; *P*<.001). However, there was no statistically significant difference in other anticoagulant effectiveness or clinical outcomes between the two groups [4]. Our previous research provided online services for patients who were taking warfarin through QQ group communication platforms, which can be accessed via smartphones, computers, laptops, and tablets. QQ is an instant messaging software service developed by Chinese company Tencent Holdings Limited. As described by You et al, social groups are some of “the main features of QQ [that allow] multiple users to communicate instantly. A message posted by a member is immediately received by all the other group members” [7]. The results showed that online services yielded similar clinical outcomes to hospital services, even though the incidence of supratherapeutic INR values increased [6]. However, there were some limitations in the QQ group communication platform. First, one patient reported his medical condition, and all the other patients could see it. There was no method of protecting patient privacy. Second, it was challenging to obtain the most recent medical information of patients because the information on all patients was mixed. Third, the information could only exist temporarily.

Recently, we developed a new warfarin dosage adjustment app named Alfalfa, which is available for installation on a mobile device; the communication between the physicians or pharmacists and the patient is point-to-point. The privacy of the patients is well protected. Meanwhile, personal medical information can be retrieved by the patients or their physicians or pharmacists at any time. As far as we know, apart from Alfalfa, there are only two studies focusing on managing warfarin with the use of mobile apps that can automatically suggest a dose and time for the next blood test based on the patient's INR value [8,9]. The difference between these mobile phone apps and Alfalfa is that Alfalfa provides a channel for communication between patients and medical staff, while the other two warfarin management apps focus on patient self-management. Alfalfa consists of a background management system and a remote anticoagulation management system. This remote anticoagulation management system can be divided into a patient terminal called Alfalfa Health Management and a medical terminal called Alfalfa Anticoagulation Guidance. The main function of Alfalfa is to facilitate the exchange of information between patients and medical staff, so that patients can report blood coagulation results and changes in their health status to physicians or pharmacists through the internet and then take their medicine and check the INR value of warfarin according to the advice of physicians or pharmacists [10]. In this study, we explored the effectiveness and safety of warfarin dose management via the Alfalfa app, which can provide further evidence in support of anticoagulant management via online anticoagulation services.

## Methods

### Study Design and Participants

We conducted a retrospective, observational cohort study to explore the effectiveness and safety of warfarin management via the Alfalfa app versus offline warfarin management. Participants were enrolled between December 2016 and March 2019 in Fujian Medical University Union Hospital (FMUUH). Inclusion criteria were patients who (1) received warfarin therapy for at least 3 months, (2) were willing to learn and accept the Alfalfa app or offline warfarin management, and (3) were willing to undergo follow-up. Exclusion criteria were pregnancy; planning to change to other anticoagulants; serious bleeding, thrombotic events, or both in the previous 3 months; and adjustment of warfarin doses by the patients themselves without a physician’s order. The primary outcome of the study, that is, percentage of time in therapeutic range (TTR), was calculated using a standard linear interpolation method [11]. Secondary outcomes included minor bleeding events, major bleeding events, thrombotic events, warfarin-related emergency department visits, warfarin-related hospital admissions, and high INR values. Major bleeding events included any bleeding requiring hospitalization or transfusion, as defined in the International Society on Thrombosis and Haemostasis classification [12]. Minor bleeding events included nose bleeding, conjunctival bleeding, gum bleeding, subcutaneous purpura, menstrual abnormalities (increased, prolonged, or advanced), and other bleeding symptoms that can be quickly stopped.

A warfarin teaching booklet was given to the patients for further reading prior to counseling as part of the standardized education process [13]. Then, the clinical pharmacist asked the patients which method of warfarin dosage adjustment would be selected after discharge: offline services or Alfalfa app. For offline services, patients could go to the local or big city hospital clinics for warfarin management. Patients were required to learn about warfarin through the booklet and complete a paper questionnaire (Multimedia Appendix 1) in wards or anticoagulation clinic. A validated questionnaire was chosen to determine the degree of anticoagulation knowledge [14]. For the Alfalfa app warfarin management, patients needed to download and install the app on their smartphones. Additionally, patients were instructed on how to use the app. Prior to registration, patients were required to learn about warfarin and complete the questionnaire. When a questionnaire score greater than or equal to 90 (out of 100) was achieved, they could register. The registration information includes name, age, body weight, anticoagulation indication, other diseases, concurrent medications, and dietary habits. Patients may select one of the physicians or pharmacists in the Alfalfa app to modify their warfarin dosage. The selected physician or pharmacist accepts the request from each patient. Patients may complete an INR test by point-of-care at home or hospital.

The protocols of warfarin dose adjustment are determined by the anticoagulation team in the FMUUH. Monitoring is frequently performed at the beginning of warfarin therapy, with at least one visit per week in the first two weeks of therapy, followed by one visit every two to four weeks. The target INR range is 1.7-2.5 for patients with valve replacement and 2.0-3.0 for those with atrial fibrillation and venous thrombosis. Our study protocol was approved by the Ethics Committee of FMUUH (2016KY036).

### Data Collection

**Figure 1:**
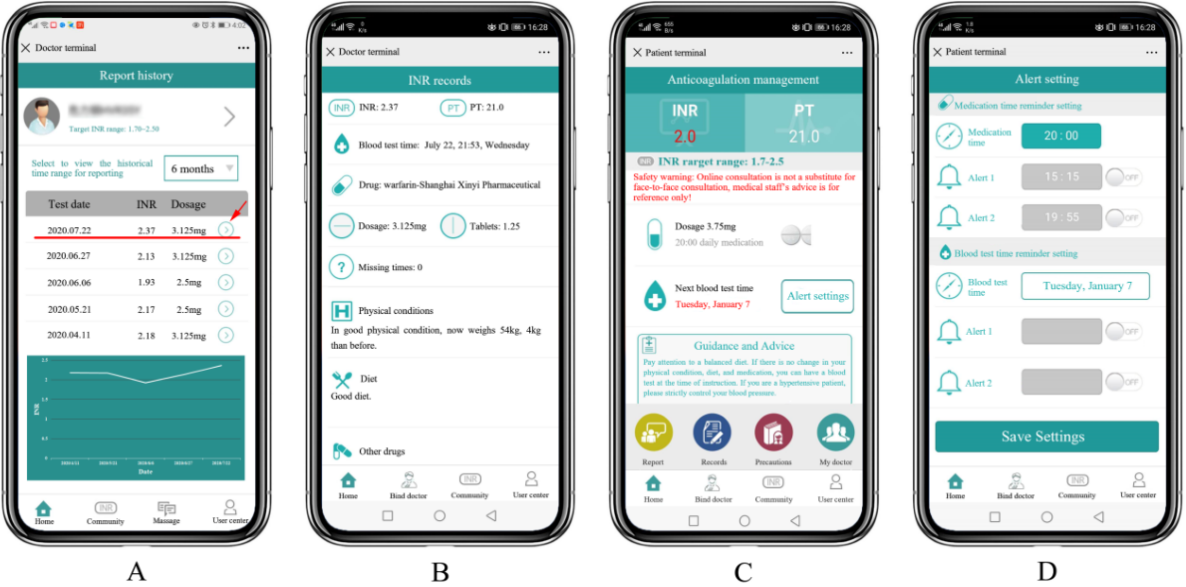
Alfalfa app.

The data automatically recorded in the Alfalfa app included demographic characteristics, indication for warfarin therapy, duration of anticoagulation, target INR range, INR at each report, warfarin dose at each report, bleeding events, thromboembolic events, emergency department visits, and hospitalization (Figure 1A and B). Patients received alerts and reminders on subsequent INR testing and time for daily warfarin doses (Figure 1C and D). When the online appointment time arrived, patients completed the INR testing and reported their INR results, warfarin dosage, disease state, concurrent medications, and dietary habits. Then, they received information on the adjusted warfarin dosage, time for subsequent INR testing, and recommendations on diet and exercise from physicians or pharmacists. This information was automatically collected in the Alfalfa app. The data from the offline warfarin management service were collected from electronic medical records and telephone follow-ups.

### Statistical Analysis

Statistical analysis was performed using Microsoft Excel, version 2019 (Microsoft Corp), and SPSS, version 20.0 (IBM Corp).

For different variables in the baseline characteristics and results, different statistical methods were used to check the differences. An independent two-sample, two-tailed *t* test was used for statistical analysis of continuous variables, including age and TTR. A chi-square test or Fisher exact test was used for categorical variables, including sex, conditions, diseases, target INR, result of efficacy, adverse events, and INR results. Continuous variables are expressed as mean (SD), and categorical variables are expressed as quantity (percentage). Statistical significance is set at *P*<.05.

## Results

### Participant Characteristics

A total of 824 patients were enrolled and assigned to either the offline warfarin management group (n=399) or the Alfalfa app group (n=425). There were no significant differences in age, sex, conditions, diseases, coprescribed antiplatelet therapy, or target INR between the groups. In the current study, 48.07% of the participants were men (Table 1). The main condition was heart valve replacement, and the main disease was hypertension.

**Table 1:** Patient characteristics.

Characteristics		Offline (n=399)	Alfalfa app (n=425)	*P* value
Age (years), mean (SD)		53.00 (12.28)	51.41 (12.54)	.07
Male sex, n (%)		175 (43.86)	237 (55.76)	.23
**Conditions, n (%)**				
	Heart valve replacement	358 (89.72)	381 (89.65)	.97
	Atrial fibrillation	22 (5.51)	22 (5.18)	.83
	Vein thromboembolism	19 (4.76)	22 (5.18)	.78
**Diseases, n (%)**				
	Hypertension	73 (18.30)	60 (14.12)	.10
	Hyperuricemia or gout	9 (2.26)	11 (2.59)	.76
	Diabetes	15 (3.76)	15 (3.53)	.86
	Co-prescribed antiplatelet therapy	7 (1.75)	8 (1.88)	.89
**Target INR^a^, n (%)**				
	1.5-2.0	107 (26.82)	126 (29.65)	.37
	1.7-2.5	271 (67.92)	277 (65.18)	.40
	2.0-3.0	21 (5.26)	22 (5.17)	.96

^a^INR: international normalized ratio.

### Anticoagulant Control and Adverse Events

The TTR was significantly higher in the Alfalfa app group than in the offline warfarin management group (79.35% vs 52.38%, *P*<.001). Furthermore, the Alfalfa app group had a higher percentage of INR values in range than the offline group (3314/4282, 77.39% vs 2005/4202, 47.72%; *P*<.001). Patients managed by the Alfalfa app had lower rates of subtherapeutic (172/4282, 4.02% vs 388/4202, 9.23%; *P*<.001), supratherapeutic (487/4282, 11.37% vs 882/4202, 20.99%; *P*<.001), and extreme subtherapeutic INR values (290/4282, 6.77% vs 910, 21.66%; *P*<.001) compared to those managed by offline services. There were similar incidences in extreme supratherapeutic INR values (19/4282, 0.44% vs 17/4202, 0.40%; *P*=.78) between the two groups (Table 2).

**Table 2:** Anticoagulant control.

INR^a^ value	Offline (n=4202)	Online (n=4282)	*P* value
Time in therapeutic range (%), mean (SD)	52.38 (12.67)	79.35 (26.31)	<.001
Extreme subtherapeutic, n (%)	910 (21.66)	290 (6.77)	<.001
Subtherapeutic, n (%)	388 (9.23)	172 (4.02)	<.001
Therapeutic, n (%)	2005 (47.72)	3314 (77.39)	<.001
Supratherapeutic, n (%)	882 (20.99)	487 (11.37)	<.001
Extreme supratherapeutic, n (%)	17 (0.40)	19 (0.44)	.78

^a^INR: international normalized ratio.

The incidences of major bleeding events (2/425, 0.5% vs 12/399, 3.0%; *P*=.005), warfarin-related emergency department visits (13/425, 3.1% vs 37/399, 9.3%; *P*<.001), and warfarin-related hospital admissions (1/425, 0.2% vs 12/399, 3.0%; *P*=.001) were lower in the Alfalfa app group than in the offline management group. However, the Alfalfa app group had a higher incidence of minor bleeding events than the offline group (45/425, 10.6% vs 20/399, 5.0%; *P*=.003). There were similar incidences in thromboembolic events (1/425, 0.2% vs 1/399, 0.3%; *P*=.53) between the two groups (Table 3).

**Table 3:** Adverse events.

Characteristics	Offline (n=399), n (%)	Alfalfa app (n=425), n (%)	*P* value
Major bleeding events	12 (3.1)	2 (0.5)	.005
Minor bleeding events	20 (5.0)	45 (10.6)	.003
Thromboembolic events	1 (0.3)	1 (0.2)	.53
Warfarin-related emergency department visits	37 (9.3)	13 (3.1)	<.001
Warfarin-related hospital admissions	12 (3.0)	1 (0.2)	.001

## Discussion

Patients taking warfarin need to monitor INR and adjust their warfarin dosages accordingly. The included patients took warfarin mainly for the following three reasons: heart valve replacement, vein thromboembolism, and atrial fibrillation. Warfarin is the only oral anticoagulant available for thrombosis prevention after heart valve replacement [15]. The type of prosthetic valve, its anatomical location, and patient-specific risks of thromboembolism and bleeding influence the specific intensity and duration of antithrombotic treatment to prevent prosthetic valve thrombosis [16]. Some studies have proved that Chinese people need lower anticoagulation intensity INR (1.5-2.5) to warfarin in comparison to the recommended INR (2.5-3.5) in developed countries [17-19]. As long-term anticoagulant therapy for vein thromboembolism, an authoritative guideline has suggested warfarin adjusted to achieve an INR of 2.0-3.0 [20]. In patients with atrial fibrillation, warfarin reduces the relative risk of stroke by 64% and all-cause mortality by 26% [21]. Clinical studies have confirmed that when INR ranges between 2.0 and 3.0 in patients with atrial fibrillation, warfarin effectively prevents stroke and does not significantly increase the risk of bleeding [22]. The effectiveness of anticoagulant therapy is usually expressed as TTR or INR within the therapeutic target range. TTR values greater than 65% are indicative of effective anticoagulation therapy [21]. Due to the different medical standards and management methods in different regions, the TTR range is wide (29%-75%) [23]. Even in strictly controlled, large-scale clinical trials such as the ROCKET AF study, the average TTR was only 55.2% for all patients, among whom Chinese patients with atrial fibrillation had a TTR of only 47%. It was challenging for anticoagulation therapy to achieve the desired effect [23,24].

In this study, TTR was significantly higher in the Alfalfa app group than in the offline warfarin management group (79.35% vs 52.38%, *P*<0.001). These results were similar to those reported by Prochaska et al, who showed that TTR was significantly higher from telemedicine-based coagulation service than from regular anticoagulation management (75.5% vs 66.3%, *P*<0.001) [25]. However, a study by Lee et al revealed opposite results [26]. Some studies have reported similar TTR between online anticoagulation management and regular medical care [27-29]. This study showed that the rates of subtherapeutic, supratherapeutic, and extreme subtherapeutic INR values were significantly lower in the Alfalfa app group than in the offline warfarin management group. The lower rate of abnormal INR distribution may reduce the incidence of bleeding and thrombotic events.

Our research findings showed that the Alfalfa app had lower incidences of major bleeding events, warfarin-related emergency department visits, and warfarin-related hospital admissions than the offline warfarin management group. However, the incidence of minor bleeding events was higher in the Alfalfa app group. This finding was similar to the results reported by Blissit et al, who concluded that the incidence of minor bleeding events was higher in the online anticoagulation management group [30]. The top three minor bleeding types in the offline group were nose bleeding (7 cases), gum bleeding (7 cases), and hematuria (4 cases). The top three minor bleeding types in the Alfalfa app were gum bleeding (10 cases), menstrual abnormalities (increased, prolonged, or advanced; 7 cases), and subcutaneous purpura (9 cases). More minor bleeding events in the Alfalfa app may be due to the way patients provided feedback. The data of the offline group are obtained through follow-up, while the data of the Alfalfa app group are actively reported by the patient in the app. This study is a retrospective study. Most patients received follow-up calls after taking warfarin for 3 months. It is possible that patients could not recall the minor bleeding adverse reactions and relay them to the investigator. On the other hand, patients in the Alfalfa app group have a stronger willingness to communicate with medical staff. Thus, the Alfalfa app showed more minor bleeding events.

The studies by Xia et al and Cryder et al showed that there were fewer anticoagulation-related hospital admissions in the online anticoagulation group [4,27]. Most studies have reported that there are no differences in the incidence of major bleeding events between the two groups [5,27-32]. Our findings revealed that the incidences of thromboembolic events were similar between the two groups. While the offline anticoagulant management models were similar among the studies, the online management models were slightly different. For example, some studies managed patients through text messages, while others relied on Bluetooth and online communication platforms. The Alfalfa app is the first app specifically designed for warfarin dose adjustment management. This study showed that the application of the app not only achieved better anticoagulant control effects, but also greatly reduced major bleeding events, emergency department visits, and hospitalization. Therefore, the Alfalfa app is likely to reduce related medical costs. There are two main reasons for the positive effects of the Alfalfa app. First, the Alfalfa app can retrieve the patient’s previous warfarin dosage, INR value, bleeding and thrombotic events, and adverse reaction history so that physicians and pharmacists can better analyze the reason for abnormal INR or bleeding. Physicians and pharmacists can assess whether the abnormal INR or bleeding events are caused by warfarin or induced by other diseases. Second, the Alfalfa app has an automatic response function for the physicians or pharmacists according to the protocols of warfarin dose adjustment. After the automatic response, physicians or pharmacists can check whether the automatic response is correct before it is submitted to the patients. This function is considerably helpful for anticoagulation management newcomers.

However, the results of this study showed that anticoagulation control may not be completely consistent with the clinical outcomes. For example, the extreme supratherapeutic INR values in this study were distributed close to each other, but the incidence of major bleeding events was lower in the Alfalfa app group. The average TTR of the offline warfarin management group was only 52.38% (<60%). In theory, the incidence of thrombosis should be higher in the offline warfarin management group, but the incidences of thrombosis were similar between the two groups.

In recent years, the internet has made considerable progress in the management of chronic diseases, especially hypertension and diabetes. The internet provides novel opportunities in anticoagulation management. Telemedicine breaks geographical and spatial limitations, providing high-quality medical resources for patients living in vast rural and remote areas. Similarly, INR real-time detection technology is convenient for at-home INR detection. This technology allows patients to enjoy high-quality online anticoagulation management services at home.

Currently, rural and medically underresourced areas often lack specialist anticoagulant physicians or pharmacists, resulting in a significant risk of bleeding and thrombosis for patients living in these areas. We are conducting a national randomized controlled multicenter study comparing the effects of the Alfalfa app and offline services [33]. If the Alfalfa app becomes widely available, there could be significant benefits for such patients. At the same time, we also look forward to the economic evaluation results of these two management models.

There were some limitations to this study. First, as a retrospective study, subjects were not assigned to either group; therefore, selection bias may be present. Second, the participants were included from a single center in Southeast China, which may not be representative of all patients with anticoagulation therapy. More prospective, randomized, and multicenter studies are required to confirm our findings.

In conclusion, warfarin management in the Alfalfa app group was superior to that in the offline group in terms of TTR, abnormal INR, major bleeding events, warfarin-related emergency department visits, and warfarin-related hospital admissions. Warfarin management via the Alfalfa app may be suitable for patients living in rural and remote areas.

## Appendix

Multimedia Appendix 1 Questionnaire.

## Abbreviations

FMUUH: Fujian Medical University Union Hospital
INR: international normalized ratio
TTR: time in therapeutic range
