# Supplementary material for: Effectiveness of the Alfalfa App in Warfarin Therapy Management for Patients Undergoing Venous Thrombosis Prevention and Treatment: Cohort Study
Source: JMIR Mhealth Uhealth. 2021 Mar 2;9(3):e23332. doi: 10.2196/23332 (PMC7967226; doi:10.2196/23332)
Supplement: Multimedia Appendix 1 [file mhealth_v9i3e23332_app1.docx]

**AKA instrument (correct answers indicated with *)**

1. Which one of these medications is recommended if you are taking Coumadin (warfarin) and want relief from a headache?

a. Advil

b. Motrin

c. Aspirin

d. Tylenol*

2. Which of the following food items would interfere with your Coumadin (warfarin) medication?

a. Bacon

b. Broccoli*

c. Bananas

d. Peeled cucumbers

3. While on Coumadin (warfarin) medication, in which of the following would you go directly to the emergency room?

a. Small bruises

b. Your appetite dramatically increases

c. Nosebleed which will not stop bleeding*

d. Gums which bleed for a few seconds after brushing teeth

4. You just remembered that you forgot to take your evening Coumadin (warfarin) medication dose last night. You would

a. skip the dose of Coumadin (warfarin) you missed*

b. take the missed Coumadin (warfarin) dose right now

c. wait and take 2 doses of Coumadin (warfarin) this evening

d. take one-half of the missed dose of Coumadin (warfarin) right now

1. While on Coumadin (warfarin) you

a. should not eat spinach

b. can eat spinach one time a month

c. can eat as much spinach as you would like whenever you would like

d. can eat spinach but need to eat the same amount regularly every week*

1. While out with friends for dinner, you have just finished your third glass of wine. This amount of alcohol consumed in a single evening will

a. cause a decrease in your INR

b. cause an increase in your INR*

c. not affect you or your Coumadin (warfarin) in any way

d. make you sick when taking Coumadin (warfarin) medication

7. While in your pharmacy, you notice multivitamins are on sale. After some thought, you decide that you may need a multivitamin. You would

a. purchase the multivitamin and begin taking it regularly

b. not take a multivitamin because it will cause a blood clot while taking Coumadin (warfarin)

c. start taking it and bring the multivitamin to your next Coumadin Clinic visit to show the pharmacist

d. purchase the multivitamin but not start taking it until you talked with the pharmacist at your Coumadin Clinic*

8. If you ran out of your prescription for your Coumadin (warfarin) you would

a. borrow Coumadin (warfarin) from a friend, as long as it is the same dose as yours

b. call and ask for refills for that day so you do not miss a dose of Coumadin (warfarin)*

c. wait until your next appointment that is just a few days away to get a new prescription

d. do nothing because you have taken Coumadin (warfarin) long enough, otherwise there would be more refills on your prescription

9. Which of the following is an effect of Coumadin (warfarin) medication that will most likely be experienced?

a. Stroke

b. Leg clot

c. Bruising*

d. Blood in the urine

10. You have a cold, which includes a runny nose and a cough. You

a. could safely take Nyquil to help get rid of the runny nose and cough

b. take your friend’s medication that he/she uses for a bad cold because he/she is also on Coumadin (warfarin) medication

c. would call the Coumadin Clinic and tell him/her you are on Coumadin (warfarin) medication and ask what you can take for your cold*

d. decide it is safer to suffer through the cold because most cold medications will interact with your Coumadin (warfarin) medication

11. When making a dental appointment while taking Coumadin (warfarin) medication, you need to remember you

a. cannot have procedures done on your teeth while taking Coumadin (warfarin)

b. must tell your dentist you are taking Coumadin (warfarin) well in advance of having any procedure done*

c. can have procedures done and there is not a need to tell the dentist about the Coumadin (warfarin)

d. can have the dental procedure done if when you arrive at your dental appointment you tell the dentist you are taking Coumadin (warfarin)

12. When the need arises to take an antibiotic (to get rid of an infection) while taking Coumadin (warfarin), you need to

a. take half of the prescribed length of therapy, and then call the Coumadin Clinic

b. refuse to take any new medication because you are taking Coumadin (warfarin)

c. wait until your next Coumadin Clinic visit and then tell the pharmacist about the antibiotic

d. call the Coumadin Clinic right away and let them know you are starting a new medication*

13. Coumadin (warfarin) works

a. in my liver to make my blood thicker

b. in my liver to make my blood thinner*

c. in my kidneys to make my blood thicker

d. in my kidneys to make my blood thinner

14. The best time of day for me to take my Coumadin (warfarin) is

a. at lunchtime

b. in the evening*

c. in the morning before breakfast

d. any time of day when I remember

15. Which of the following is an effect of my Coumadin (warfarin) medication that I will most likely experience if my INR is too high?

a. A clot in the leg

b. Minor bleeding*

c. Clot in the lung

d. Bleeding in the brain

16. Which of the following drinks can decrease the effectiveness of your Coumadin (warfarin)?

a. Deans 2% low-fat milk

b. Hershey’s chocolate shake

c. Tropicana orange juice

d. Ensure nutritional supplement*

17. While taking Coumadin (warfarin), which of the following represents

a situation when you should to go to the emergency room?

a. You cough up blood*

b. Your nose bleeds slightly while blowing it

c. You gums bleed after brushing your teeth then it stops quickly

d. You have cut yourself while shaving and you control the bleeding

18. Your neighbor brings over this great ‘‘all natural’’ herbal supplement she just bought from her chiropractor. She swears that this helps all her aches and pains and recommends that you take it when you ache. Your decision is to

a. take her advice, realizing that you could use this herbal supplement

b. start taking the herbal supplement and tell your pharmacist at the next office visit

c. ask your pharmacist if the herbal supplement will interact with your medications before you take it*

d. avoid taking herbal supplements altogether because all medications interact with Coumadin (warfarin)

19. Once you have reached a stable Coumadin (warfarin) dose, a PT/INR blood test

a. should be checked once a year

b. should be checked once every 3 months

c. should be checked at least once every 4 weeks*

d. does not need to be checked once you are on a stable Coumadin (warfarin) dose

20. The results of your PT/INR test tells the pharmacist

a. how thick or thin your blood is while taking Coumadin (warfarin)*

b. how well your kidneys are working since taking Coumadin (warfarin)

c. what your average blood sugar level was since taking Coumadin (warfarin)

d. how much alcohol you have been drinking since taking Coumadin (warfarin)

21. While taking Coumadin (warfarin), you should call your Coumadin Clinic when you get:

a. a backache

b. an upset stomach

c. a tension headache

d. diarrhea for more than 1 day*

22. While on Coumadin (warfarin) you need to be routinely monitored for which of the following:

a. PT/INR tests*

b. Potassium levels

c. Blood glucose levels

d. Kidney function tests

23. Which of the following may have a significant effect on how well your Coumadin (warfarin) works?

a. Changes in your mood

b. Changes in sleep habits

c. How much water you drink

d. Using over the counter medications*

24. While taking Coumadin (warfarin), which of the following should lead you to the emergency room?

a. Loss of appetite

b. Brown loose stools

c. Urine becomes red in color*

d. A quarter size bruise on your arm

25. Which of the following foods could affect how well your Coumadin (warfarin) works?

a. Celery

b. Carrots

c. Cole slaw*

d. Green beans

26. You have generic and brand Coumadin (warfarin) tablets at home that are both the same dose. You should

a. take both because they work differently

b. take only brand or only generic, but not both*

c. not take either until you call the Coumadin Clinic

d. alternate days by taking brand on one day and generic on the next day

27. Once your Coumadin (warfarin) is stopped, how long does it take to get the medication to get out of your system?

a. 5 hours

b. 5 days*

c. 5 weeks

d. 5 months

28. After starting Coumadin (warfarin), how long (in months/years) would you expect to be taking Coumadin (warfarin)?

a. 1 year

b. 1 month

c. It depends on each person’s needs*

d. If you start Coumadin (warfarin), you will have to be on the medication for the rest of your life

29. Which of the following activities are more risky while taking Coumadin (warfarin)?

a. Playing football, because you can hit your head*

b. Taking a bath, because soap interacts with Coumadin (warfarin)

c. Playing cards because using your hands a lot will cause a blood clot

d. Walking a lot, because exercise is not good for you while taking Coumadin (warfarin)
